# *Chrysosplenium sangzhiense* (Saxifragaceae), a new species from Hunan, China

**DOI:** 10.3897/phytokeys.176.62802

**Published:** 2021-04-16

**Authors:** Long-Fei Fu, Tian-Ge Yang*, De-Qing Lan, Fang Wen, Hong Liu

**Affiliations:** 1 Guangxi Key Laboratory of Plant Conservation and Restoration Ecology in Karst Terrain, Guangxi Institute of Botany, Guangxi Zhuang Autonomous Region and Chinese Academy of Sciences, Guilin 541006, China Guangxi Institute of Botany, Chinese Academy of Sciences Guilin China; 2 College of Life Sciences & Key Laboratory for Protection and Application of Special Plant Germplasm in Wuling Area of Hubei Province, South-Central University for Nationalities, Wuhan 430074, Hubei Province, China South-Central University for Nationalities Wuhan China

**Keywords:** *
Chrysosplenium
*, cytology, phylogeny, Saxifragaceae, subgen, taxonomy

## Abstract

*Chrysosplenium sangzhiense* Hong Liu, a new species from Hunan, China, is described and illustrated. The phylogenetic analysis revealed that the new species belongs to subgen. Chrysosplenium and is closely related to *C. grayanum*, *C. nepalense* and *C. sinicum*. The chromosome number of the new species is 2n = 46, indicating a novel basic number x = 23 in *Chrysosplenium* that is different from other species. This also suggests that *C. sangzhiense* is probably an allopolyploid derivative of a species with x = 11 and one with x = 12. Morphologically, *C. sangzhiense* can be easily distinguished from *C. grayanum*, *C. nepalense*, *C. sinicum* and *C. cavaleriei*, a species not included in our phylogenetic analysis by a suite of characters relating to the sterile shoots, basal leaves, cauline leaves, flowering stem, sepals, disc, capsule and seed. A global conservation assessment is performed, and classifies *C. sangzhiense* as Least Concern (LC).

## Introduction

*Chrysosplenium* L. (1753) is a perennial herbaceous genus in Saxifragaceae and comprises more than 70 species (Kim et al. 2019; Fu et al. 2020). *Chrysosplenium* is distributed in Asia, America and Europe (Pan and Ohba 2001; Soltis 2007).

The latest revision of Chinese *Chrysosplenium* included 35 species (Pan and Ohba 2001). Although no particular infra-generic classification was adopted in this revision, use of leaf arrangement as the primary character in the key to species reflected the recognition of two subgenera in previous taxonomic revisions (Pan 1986a, b). In addition, seed surface has been used as an important character to delimit sections (Pan 1986a, b). Soltis et al. (2001) showed that the two subgenera are both monophyletic and sister to each other using *matK* sequence data, thereby confirming that leaf arrangement is a phylogenetically informative morphological character. Subsequent taxonomic research on Chinese *Chrysosplenium* has been undertaken by Liu et al. (2016), Kim et al. (2019) and Fu et al. (2020), bringing the total diversity of the Chinese flora to 38 species, of which 23 (60%) are endemic.

Previous studies have demonstrated that *Chrysosplenium* has a diverse basic chromosome number with x = 7, 8, 9, 10, 11, 12 and 13 at species level indicating cytological data provides important evidence for the delimitation and evolution of *Chrysosplenium* (Hara and Kurosawa 1963; Funamoto and Tanaka 1988a, b, 1989; Funamoto et al. 1997, 1999, 2000, 2004; Funamoto and Zhou 2010).

As part of ongoing research into the diversity of Chinese *Chrysosplenium*, the authors undertook an extensive fieldtrip in Hunan, China. During the trip an unknown species of *Chrysosplenium* was collected. Following a thorough literature survey (Hara 1957; Pan 1992; Pan and Ohba 2001; Liu et al. 2016; Kim et al. 2019; Fu et al. 2020) along with the molecular and cytological evidence, we confirmed that it is a distinct and undescribed species.

## Materials and methods

### Morphology observations and conservation assessments

All morphological characters were studied based on the material from field and herbarium specimens using a dissecting microscope (SMZ171, Motic, China). For seed morphology, we also undertook scanning electron micrograph (SEM) observation; seeds were collected from the field and dried by silica gel. The pre-treatment including impurities removing, air-drying and gold-coating was performed, following Fu et al. (2020). Observations and photographs were taken under a Hitachi SU8010 scanning electron microscope. At least 15 seeds were used to determine the size and surface. Conservation assessment was undertaken following IUCN (2019).

### Genomic DNA extraction, PCR amplification, and Sequencing

To confirm the systematic position of this unknown species, we conducted phylogenetic studies using *matK* sequence data. We chose this DNA region due to its highest species coverage within the genus (Soltis et al. 2001; De Vere 2012; Saarela et al. 2013; Ebersbach et al. 2017; Kim et al. 2018) so that we could trace the most closely related species. Forty-eight species of *Chrysosplenium* as in-group and three species of *Saxifraga* and *Itea* as out-group were sampled. Of these, 15 sequences were obtained from the Genbank (https://www.ncbi.nlm.nih.gov/), while 36 sequences were newly generated. Their species names and GenBank accession numbers are listed in Table 1. DNA extraction, PCR amplification, and sequencing were performed following Soltis et al. (2001).

**Table 1.** Species names and GenBank accession numbers of *matK* DNA sequences used in this study (* newly generated sequences).

Species	Location	Voucher specimens	Herbarium	Genbank number
*Chrysosplenium album* Maxim.	Nikkou-shi, Japan	HSN09815	HSN	MW402998 ^*^
*Chrysosplenium aureobracteatum* Y.I.Kim & Y.D.Kim	Gangwon, South Korea	KYI-2009032	–	AXY64019
*Chrysosplenium biondianum* Engl.	Shanxi, China	HZ2017050107362	HSN	MW402999 ^*^
*Chrysosplenium carnosum* Hook.f. et Thoms.	Sichuan, China	HSN013113	HSN	MW403000 ^*^
*Chrysosplenium davidianum* Decne. ex Maxim.	Sichuan, China	HSN06442	HSN	MW403001 ^*^
*Chrysosplenium delavayi* Franch.	Hunan, China	SZ2016080907105	HSN	MW403002 ^*^
*Chrysosplenium echinus* Maxim.	Nikkou-shi, Japan	HSN09817	HSN	MW403003 ^*^
*Chrysosplenium fauriae* Franch.	Nikkou-shi, Japan	HSN09823	HSN	MW403004 ^*^
*Chrysosplenium flagelliferum* Fr. Schmidt.	Nikkou-shi, Japan	HSN09816	HSN	MW403005 ^*^
*Chrysosplenium forrestii* Diels	Nikkou-shi, Japan	HSN7797	HSN	MW403006 ^*^
*Chrysosplenium giraldianum* Engl.	Sichuan, China	JZ2018042507981	HSN	MW403007 ^*^
*Chrysosplenium glossophyllum* Hara	Sichuan, China	QCS2017102608035	HSN	MW403008 ^*^
*Chrysosplenium grayanum* Maxim.	Nikkou-shi, Japan	HSN09810	HSN	MW403009 ^*^
*Chrysosplenium griffithii* Hook.f. et Thoms.	Shanxi, China	HSN7760	HSN	MW403010 ^*^
*Chrysosplenium henryi* Franch.	Hunan, China	HSN7505	HSN	MW403011 ^*^
*Chrysosplenium hydrocotylifolium* Lévl. et Vant.	Hubei, China	HSN09188	HSN	MW403012 ^*^
*Chrysosplenium japonicum* (Maxim.) Makino	Zhejiang, China	HSN7909	HSN	MW403013 ^*^
*Chrysosplenium kamtschaticum* Fisch. ex Seringe	Shimane-ken, Japan	DG2019032310004	HSN	MW403014 ^*^
*Chrysosplenium kiotense* Ohwi.	Nikkou-shi, Japan	HSN09818	HSN	MW403015 ^*^
*Chrysosplenium lanuginosum* Hook.f. et Thoms.	Anhui, China	BD2017030507343	HSN	MW403016 ^*^
*Chrysosplenium lectus-cochleae* Kitagawa	Jilin, China	HSN7379	HSN	MW403017 ^*^
*Chrysosplenium macrophyllum* Oliv.	Hubei, China	BD2017030507344	HSN	MW403018 ^*^
*Chrysosplenium macrospermum* Y.I.Kim & Y.D.Kim	Jilin, China	CBS2016062406656	HSN	MW403019 ^*^
*Chrysosplenium macrostemon* Maxim. ex Franch. et Sav.	Nikkou-shi, Japan	HSN09820	HSN	MW403020 ^*^
*Chrysosplenium nepalense* D.Don	Yunnan, China	GLGH20170607375	HSN	MW403021 ^*^
*Chrysosplenium nudicaule* Bunge	Gansu, China	HSN07772	HSN	MW403022 ^*^
*Chrysosplenium pilosum* Maxim.	Nikkou-shi, Japan	HSN09819	HSN	MW403023 ^*^
*Chrysosplenium qinlingense* Z.P.Jien ex J.T.Pan	Sichuan, China	HSN7980	HSN	MW403024 ^*^
*Chrysosplenium ramosum* Maxim.	Jilin, China	SJH2017052107372	HSN	MW403025 ^*^
*Chrysosplenium serreanum* Hand.-Mazz.	Jilin, China	SJH2017052107371	HSN	MW403026 ^*^
*Chrysosplenium sinicum* Maxim.	Hunan, China	TPS2017042407504	HSN	MW403027 ^*^
*Chrysosplenium taibaishanense* J.T.Pan	Shanxi, China	HSN7761	HSN	MW403028 ^*^
*Chrysosplenium uniflorum* Maxim.	Tibet, China	HSN7380	HSN	MW403029 ^*^
*Chrysosplenium zhouzhiense* Hong Liu	Shanxi, China	HSN13356	HSN	MW403030 ^*^
*Chrysosplenium sangzhiense* Hong Liu sp. nov.	Hunan, China	TPS2017042307449	HSN	MW403032 ^*^
*Chrysosplenium alternifolium* L.	Shimane-ken, Japan	DG2019032310003	HSN	MT362050
*Chrysosplenium maximowiczii* Franch. et Sav.	Kanagawa, Japan	–	–	AB003053
*Chrysosplenium nagasei* Wakab. & H.Ohba	Gifu, Japan	–	–	AB003054
*Chrysosplenium rhabdospermum* Maxim.	Nagasaki, Japan	–	–	AB003058
*Chrysosplenium tosaense* Makino	Saitama, Japan	–	–	AB003059
*Chrysosplenium iowense* Rydb.	Iowa, USA	–	–	L34120
*Chrysosplenium oppositifolium* L.	Wales, UK	–	–	JN894973
*Chrysosplenium rosendahlii* Packer	Northwest Territories, Canada	–	–	KC474470
*Chrysosplenium tetrandrum* (N. Lund) Th. Fries	Nunavut, Canada	Brysting_01-065_CAN	CAN	KC474473
*Chrysosplenium wrightii* Franch. & Sav.	Yukon, Canada	Bennett_08-125_CAN	CAN	KC474474
*Chrysosplenium americanum* Schwein. ex Hook.	Hatfield, New Hampshire, USA	–	–	KU524206
*Chrysosplenium valdivicum* Hook.	Chile	–	–	KU524208
*Chrysosplenium zhangjiajieense* X.L.Yu, Hui Zhou & D.S.Zhou	Hunan, China	ZJ2016031506369	HSN	MW402997 ^*^
*Saxifraga stolonifera* Curt.	Anhui, China	HSN07355	HSN	MW403031*
*Itea chinensis* C.K.Schneider	–	–	–	NC_037884
*Itea virginica* L.	–	–	–	MF350096

### Phylogenetic analysis

We performed phylogenetic analyses of *Chrysosplenium* based on *matK* sequence dataset using Bayesian inference (BI) and maximum likelihood (ML). For BI analysis, we employed MrBayes v.3.2.6 (Ronquist et al. 2012) to obtain a maximum clade credibility (MCC) tree. The matrix of *matK* sequence was aligned by MAFFT. Bayesian inference was performed using one million generations, four runs, four chains, a temperature of 0.001, 25% trees discarded as burn-in, and trees sampled every 1,000 generations (1,000 trees sampled in total) with GTR+F+G4 model.

We conducted the ML analysis using IQ-TREE v 2.0.6 (Nguyen et al. 2015) with 1,000 bootstrap replicates, and default ModelFinder (Kalyaanamoorthy et al. 2017) to find TVM+F+R3 as the best-fit substitution model. Tree visualization was achieved in FigTree v1.4.3 (http://tree.bio.ed.ac.uk/software/figtree/).

### Chromosome preparations

Living plants of the new species were cultivated in the green house of South-Central University for Nationalities. Actively growing root tips were harvested after 1–2 weeks. Cytological examination was performed following Funamoto and Zhou (2010). The best metaphase plates were photographed using an imager microscope with a camera attachment. At least 3–5 cells from 3–5 root tips of five individuals of the new species at somatic metaphase were counted to determine the chromosome numbers.

## Results

### Molecular phylogenetic studies

**Figure 1.**
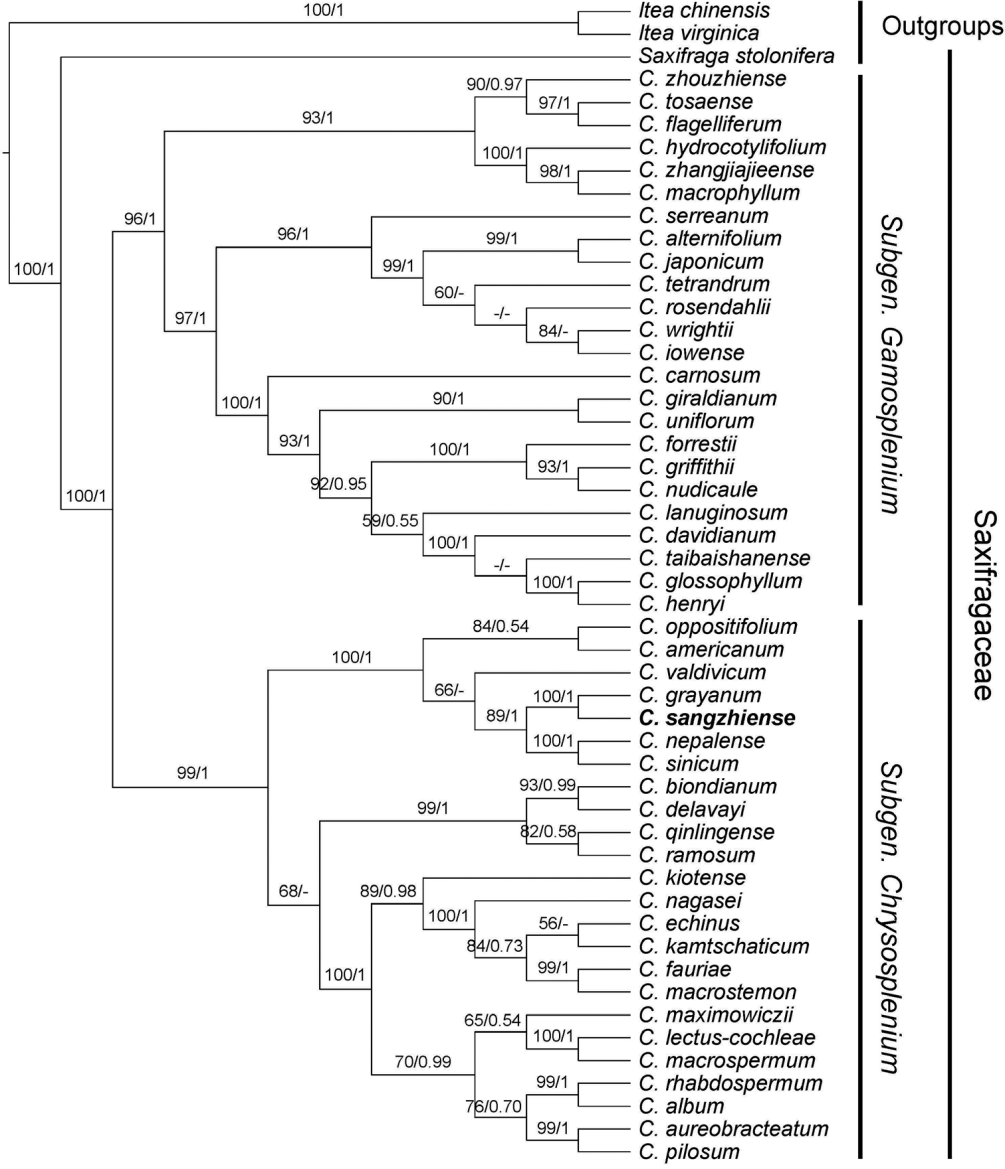
Phylogenetic tree of *Chrysosplenium* generated from maximum likelihood (ML) of *matK* dataset. Numbers on the branches indicate bootstrap values (≥50%) of the ML and the posterior probability (≥0.5) of Bayesian inference analyses.

The aligned matrix of *matK* sequence was 1,644 characters. Of the 154 variable characters, 90 (58.44%) were parsimony-informative, including indels. BI and ML analyses resulted in the same tree topology which showed the undescribed species as belonging to a strongly supported clade (BP = 89%, PP = 1) that included *Chrysosplenium grayanum* Maxim. (1877), *C. nepalense* D.Don (1825) and *C. sinicum* Maxim. (1877) (Fig. 1).

### Chromosome characteristics

**Figure 2.**
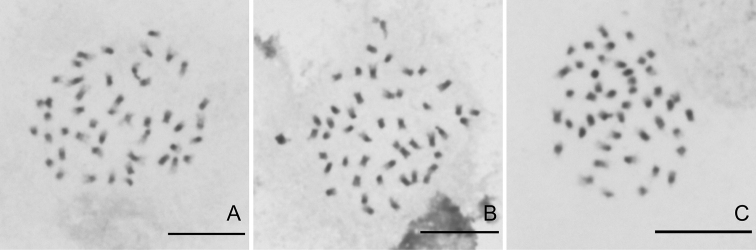
Somatic chromosomes at metaphase of *C. sangzhiense* Hong Liu, sp. nov. from three different individuals. Scale bar: 10 μm.

The chromosome number of *Chrysosplenium sangzhiense* was observed to be 2n = 46 (Fig. 2). The chromosome size fell into the range 0.93–2.43 μm, suggesting slight size variation. A detailed karyotype analysis was not possible because the chromosomes are small, and the position of centromere could not be determined.

### Taxonomic treatment

#### Chrysosplenium sangzhiense

Taxon classificationPlantaeSaxifragalesSaxifragaceae

Hong Liu sp. nov.

7D5FF645-2A2F-55D7-96FB-7EF1109D1244

urn:lsid:ipni.org:names:77216564-1

Figs 3 , 4 , 5

##### Remarks.

Similar to *Chrysosplenium grayanum*, *C. nepalense*, *C. sinicum* and *C. cavaleriei* (Table 2). *C. sangzhiense* differs from *C. grayanum* in its usually fewer cauline leaves, a square flowering stem and red-brown seeds; from *C. nepalense* it differs in its usually fewer cauline leaves, a square flowering stem and conspicuously unequal capsule lobes; from *C. sinicum* it differs in producing sterile shoots from all leaf axils, an absence of basal leaves, larger cauline leaves, and red-brown seeds; and from *C. cavaleriei* it differs in its erect sepals and absent disc.

**Figure 3.**
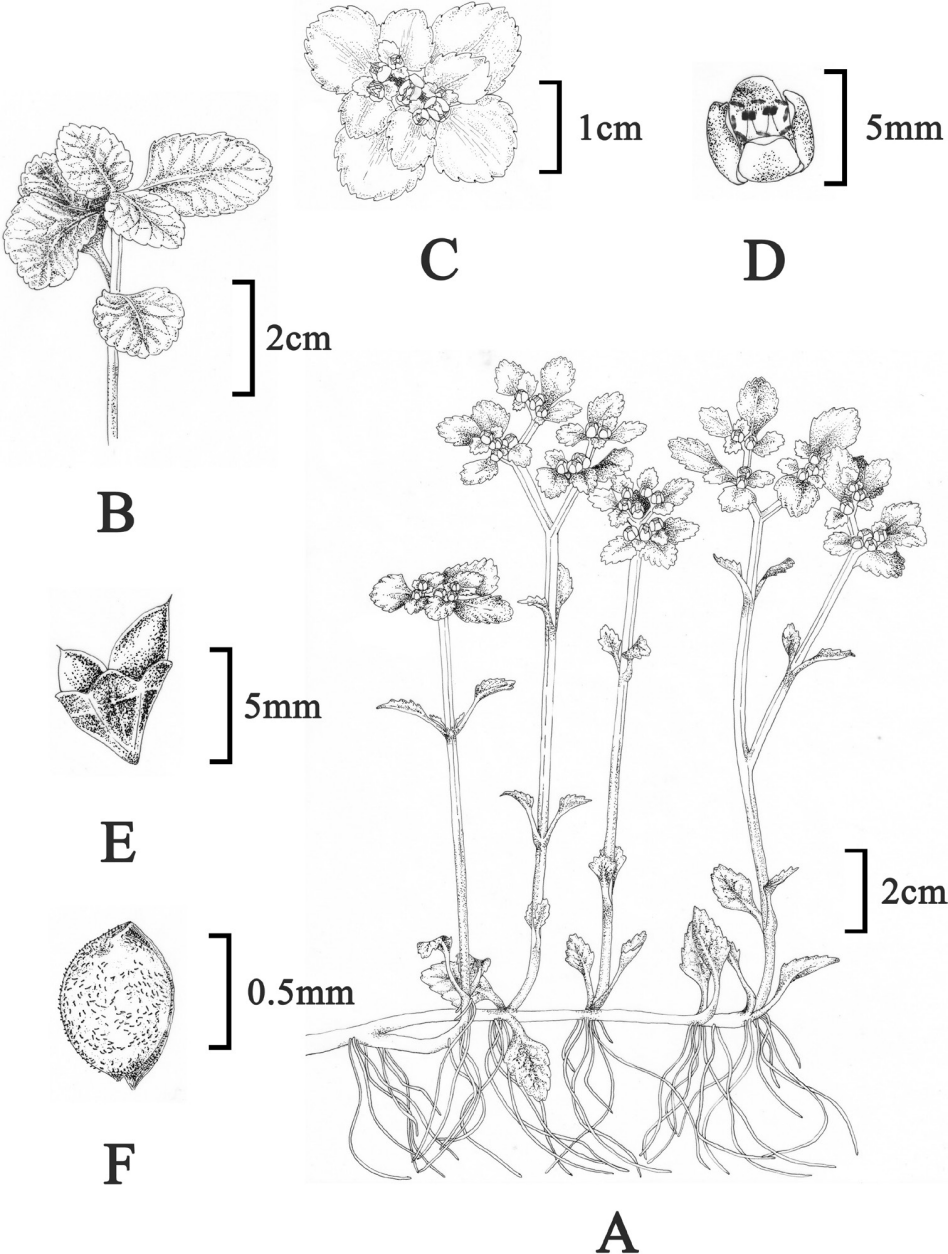
Illustration of *Chrysosplenium sangzhiense* Hong Liu, sp. nov. **A** habit in flowering phase **B** non-flowering stem **C** inflorescence with flowers **D** flower **E** capsule **F** seeds.

**Table 2.** Morphological comparison of *Chrysosplenium sangzhiense*, *C. cavaleriei*, *C. grayanum*, *C. nepalense* and *C. sinicum*.

Characters	*C. sangzhiense*	*C. cavaleriei*	*C. grayanum*	*C. nepalense*	*C. sinicum*
**Sterile branch**	from all leaf axils	from near stem base	from all leaf axils	from all leaf axils	only from basal leaf axils
**Basal leaves**	absent	absent	absent	absent	present
**Cauline leaves**	2–3 pairs, 10–30 × 10–25 mm	1–3 pairs, 9–13 × 10–14 mm	2–7 pairs, 4–17 × 4–17 mm	3–5 pairs, 3–18 × 5–18 mm	1–2 pairs, 6–10.5 × 7.5–11.5 mm
**Flowering stem (upper part)**	square	unknown	rounded	rounded	square
**Sepals**	erect	spreading	erect	erect	erect
**Disc**	absent	distinct	somewhat inconspicuous	absent	absent
**Capsule lobe**	lobes conspicuous unequal	lobes conspicuous unequal	lobes conspicuous unequal	lobes subequal	lobes conspicuous unequal
**Seed**	red brown, papillose	dark brown, papillose	dark brown, papillose	red brown, smooth	dark brown, papillose

##### Type.

China. Hunan: Badagongshan National Nature Reserve, Sangzhi County, 29°47'10"N, 110°5'33"E, under broadleaved forests and near the stream in a mountain area at ca 1,220 m altitude, 22 April 2017, *Hong Liu HSN07449* (holotype HSN; isotypes HSN, IBK).

##### Description.

Perennial herbs, 10–25 cm tall. ***Root*** fibrous and robust. ***Rhizome*** long creeping without stolons or bulbs. ***Basal leaves*** absent. ***Sterile shoots*** well developed, arising from all leaf axils, round in cross-section, 5–15 cm long at anthesis, later elongate and decumbent, up to 50 cm long, rooting at nodes, without forming a rosette. ***Leaves*** of sterile shoots opposite, isophyllous, always ca 8 at anthesis, dark purple, petiole 6–10 mm long, blade 10–30 × 10–25 mm, rounded, glabrous, apex obtuse, margin obtusely dentate (10–16 teeth), base broadly cuneate; post-anthesis 10–30 or more, green, petiole 6–10 mm long, blade 20–35 × 15–20 mm, rounded or ovate, glabrous, apex obtuse, margin obtusely dentate (12–20 teeth), base broadly cuneate. ***Cauline leaves*** 4–6 (2–3 pairs), opposite, petiole 6–10 mm long; blade 6–13 × 5–12 mm, rounded or broadly ovate, glabrous, apex obtuse, margin obtusely dentate (10–14 teeth), base broadly cuneate. ***Flowering stem(s)*** erect, branched, 10–23 cm tall, glabrous, purple, square in cross-section. ***Inflorescence*** 8–25-flowered cyme, dense, 1.4–9 cm long, 5–10 cm in diam.; ***bracteal leaves*** yellow-green, triangular arrangement and unequal, the middle one larger, petiole 2–8 mm long, blade 4–15 × 7–10 mm, subrounded, glabrous, apex obtuse, margin obtusely dentate (6–12 teeth), base broadly cuneate; ***Flowers*** tetramerous, actinomorphic; ***sepals*** 4 (2 pairs), erect, yellow in flowering phase but turn green in fruiting time, 2–3 × 2–3 mm, broadly ovate, apex obtuse; disk absent; ***stamens*** 8, homostylic, 1–2 mm long, shorter than sepals; filaments slender, ca 1 mm long; anther yellow, 2-locular, longitudinally dehiscent; ovary 2-locular, semi-inferior; stigma 2; styles erect, ca 1–2 mm long. ***Fruit*** a capsule, 5–7 mm long, green, smooth, 2-lobed (horn-shaped), conspicuous unequal, dehiscent along the adaxial suture; seeds numerous, reddish brown, sub-ovoid, a raphe on one side, 650–800 × 600–750 μm, papillose.

**Figure 4.**
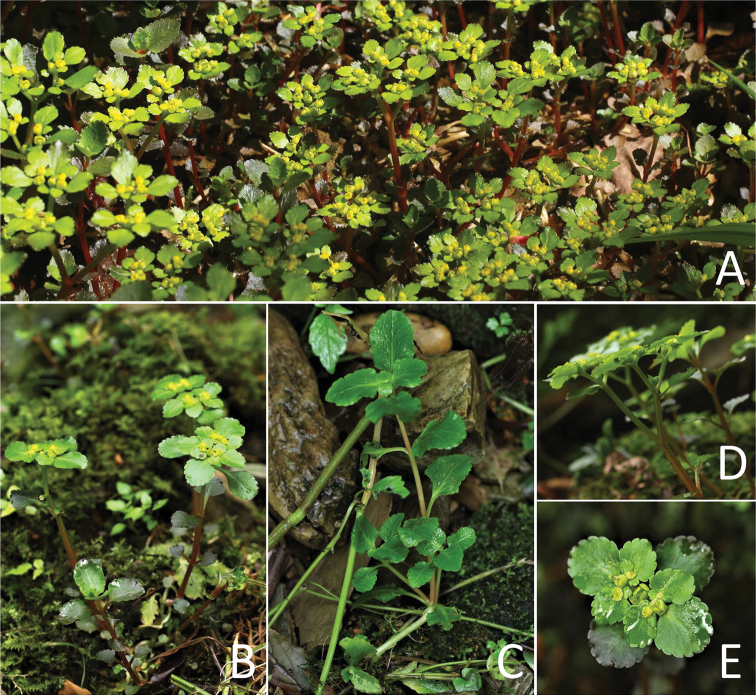
Plate of *Chrysosplenium sangzhiense* Hong Liu, sp. nov. **A** habitat **B** habit in flowering phase **C** habit in non-flowering phase **D** flowering stem **E** inflorescence with flowers (Photos by Hong Liu).

##### Etymology.

*Chrysosplenium sangzhiense* is named after the type locality, Sangzhi County, Hunan Province, China.

##### Vernacular name.

sāng zhí jīn yāo (Chinese pronunciation); 桑植金腰 (Chinese name).

##### Conservation status.

At present, *Chrysosplenium sangzhiense* is only known from a single locality (IUCN criterion D2). At this locality, the population is ca 500 mature individuals (IUCN criterion D1) growing in at least ten patches within a nature reserve. Using the IUCN methodology, *C. sangzhiense* would be classed as Vulnerable (VU), however no plausible threat could be found to confirm its status as the population is located within a protected area and not under threat in the near future. In addition, considering that the surrounding area has not been completely explored, there may be hitherto undocumented additional populations. For these reasons the Global Species Conservation Assessment for *C. sangzhiense* is Least Concern (LC).

**Figure 5.**
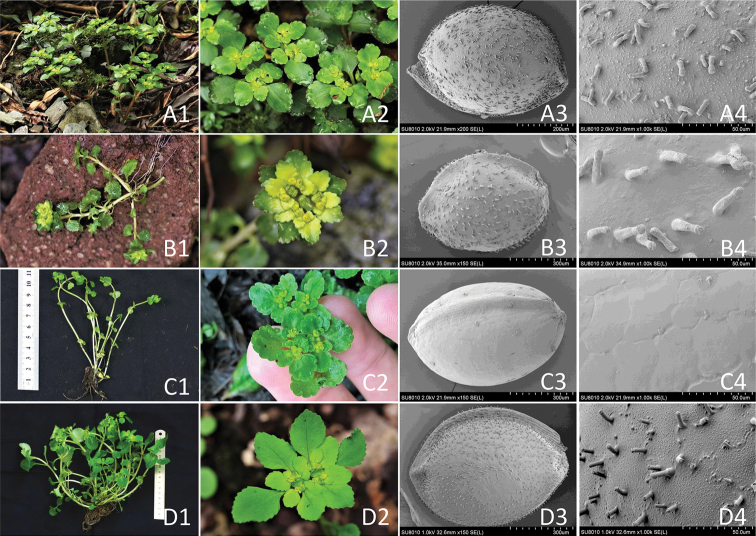
Macro- and micro-morphology of *Chrysosplenium* spp. **A***C. sangzhiense*, habit (**A1**), inflorescence with flowers (**A2**), scanning electron micrograph (SEM) of seed 200× **(A3)**, 1,000× **(A4) B***C. grayanum*, habit (**B1**), inflorescence with flowers (**B2**), SEM of seed 150× (**B3**), 1,000× (**B4**) **C***C. nepalense*, habit (**C1**), inflorescence with flowers (**C2**), SEM of seed 150× (**C3**), 1,000× (**C4**) **D***C. sinicum* habit (**D1**), inflorescence with flowers (**D2**) SEM of seed 150× (**D3**), 1,000× (**D4**) (Photos by Hong Liu).

## Discussion

Our phylogenetic analysis is consistent with previous studies (Soltis et al. 2001) that *Chrysosplenium* is monophyletic and comprises two strongly supported clades namely subgen. Gamosplenium (with alternate leaves) and subgen. Chrysosplenium (with opposite leaves). *C. sangzhiense* is recovered as a member of subgen. Chrysosplenium and falls into a strongly supported clade that includes *C. grayanum*, *C. nepalense* and *C. sinicum*. In addition, *C. cavaleriei* H.Lév. & Vaniot (1911) is also a morphologically similar species despite that it is not included in our phylogenetic analysis. All five species are close morphologically (Table 2), but nevertheless distinguishable. *C. grayanum* is likely the most closely related species despite the fact that it is endemic to Japan, while *C. nepalense*, *C. sinicum* and *C. cavaleriei* are widespread in China.

The basic chromosome number of Japanese *Chrysosplenium* species is x = 11 or x = 12, but in China there is more diversity with x = 7, 8, 9, 10, 12 and 13 (Hara and Kurosawa 1963; Funamoto and Tanaka 1988a, b, 1989; Funamoto et al. 1997, 1999, 2000, 2004; Funamoto and Zhou 2010). Our cytological studies support this. The chromosome number of *C. sangzhiense* is 2n = 46 indicating its basic number to be x = 23. Given the relationship of reported basic chromosome number of *Chrysosplenium*, it suggests that the new species is probably an allopolyploid derivative of a species with x = 11 and one with x = 12. Furthermore, this is a novel basic number for the genus, and different from the closely related species such as *C. grayanum* (x = 11), *C. sinicum* (x = 12) and *C. nepalense* (x = 12) (Hara and Kurosawa 1963; Funamoto and Tanaka 1989; Funamoto et al. 1999; Funamoto and Zhou 2010).

## Conclusion

In this study, we confirm and describe a new species of *Chrysosplenium* based on morphological, molecular and cytological evidence. The newly generated molecular data contributes to reconstruct a robust phylogenetic framework for further studies on the aspects of biogeography and character evolution of *Chrysosplenium*. In addition, a novel basic chromosome number for *Chrysosplenium* reported here will be useful data to evaluate the evolutionary pattern of chromosome number change and to estimate the basic chromosome number of clades of the genus.

## Supplementary Material

XML Treatment for Chrysosplenium sangzhiense
